# Impressions of Cuba and the Cubans1Read before the Buffalo Academy of Medicine, May 2, 1899.

**Published:** 1899-06

**Authors:** Marshall Clinton

**Affiliations:** Buffalo, N. Y., Late Assistant Surgeon 202d New York Volunteers.


### IMPRESSIONS OF CUBA AND THE CUBANS.[1]

By MARSHALL CLINTON, M. D., Buffalo, N. Y.,
Late Assistant Surgeon 202d New York Volunteers.

AFTER nine months' service as assistant surgeon in the 202d N. Y. Vols., I have the pleasure of presenting to you a few impressions gathered by the way, of Cuba, Cubans and the work of and dangers to United States troops stationed at that island. Cuba is naturally a beautiful spot and if the climatic conditions were in keeping with the scenery it would be a veritable paradise. The temperature rarely goes above 90° F. or below 60° F. Humidity is the cause of all the trouble, and combined with a burning sun, the powerful rays of which feel like the heat shafts from a red hot furnace, furnishes from decomposing materials, pabulum for those essential dirt diseases with which Cuba is infected.

The Cubans are not a hardy, healthy looking race, if I may call them a race. They are the union of North American Indian, Spanish and negro blood and have the marks of degeneration plainer and more common than one sees among our own people. Usually slight in build, lacking color and strength, and by nature lazy and irresponsible, they do not stand continued fatigue and lack totally that open-hearted frankness and sturdy carriage of our Northern soldiers. They possess an acquired immunity to yellow fever, having, as a rule, had a slight attack during childhood. This immunity persists

1. Read before the Buffalo Academy of Medicine, May 2, 1899.

as long as they remain in good health and live in Cuba, but residence in a temperate climate for three years or more renders them liable to reinfection on their return. During the beginning and end of the rainy season they are liable to attacks of intermittent malarial fever, which yield, however, very readily to treatment.

Sanitation is to them an unknown science and the conditions under which they live are revolting. They are not cleanly in body or habit. The typical Cuban dwelling is a stone affair, one or two stories high, built in a square or "L" shape, and including a court-yard. The living rooms all open into this and in one corner is the kitchen and privy, separated by a slight partition, but forming one room. The privy vault, two feet wide, four feet long and from four to six feet deep is lined with a rough tiling, permitting the fluids to ooze through into the surrounding ground. On a level with the street is a drain-pipe to carry any over-flow to the gutter outside. The poorer houses have no privies and the make shift is a commode, the accumulated contents of which are thrown into the street after dark. Kitchen refuse is disposed of by being thrown into the street, where a hot, damp atmosphere and a burning sun quickly obliterate it. In the abandoned Spanish military hospital was a tubercular ward and on the stone floor beside spots where cots had evidently been placed was over half an inch of solid dried sputum. This same hospital is on the edge of the town, a short distance from and draining into the brook that supplies the town with water. It was a common sight when riding along the small stream to see a negro taking a bath near the spring that fed the brook; several hundred feet below a woman doing the household laundry on a flat rock, and still further down see people filling pails with the same water for household purposes. There were many cases of chronic starvation among the poor natives upon our arrival, the result of the reconcentrado régime. The civil authorities estimate that during the last three years more than one-third of the entire population had died as the result of warfare and starvation. While the Cuban soil is marvelously productive, the natives being able in forty days to raise a crop of garden truck, yet the reconcentradoes did not begin until lately to plant the soil, as they had neither seeds, tools, nor ambition. Among these people were found the clinical pictures of chronic starvation, emaciated, spindle-arms and legs, pinched, wan, bloodless faces, hollow chests with the ribs almost bursting through the skin, sores on legs and faces, large protuberant abdomens, from fatty livers, enlarged spleens and ascities. For three years their diet has been vegetables and sugar-cane, the latter forming their principal food stuff.

Some Cuban soldiers removed from their barracks in the mountains to the civil hospital were suffering from this disease. One died from pneumonia and three others from renal insufficiency, a short time after their arrival. According to the statements of the local physicians the lessened vitality rendered them liable to disease that healthy natives are usually immune to. The average Cuban dies of old age or the fibroses of old age at from 50 to 60. A man 65 years old is a patriarch. As age advances they rarely grow obese, but become thin and shriveled, and at 45 one sees men older looking than men here at 60.

On the evacuation of the towns by the Spaniards the Cubans emerged from their hiding places in the hills and there were many opportunities to observe among them the effect of the Mauser bullet. One Cuban major was particularly interesting, as during various potshot engagements he had accumulated three wounds of the right chest, four of the right thigh, and two in the left forearm. These had all healed with very little trouble other than the incident shock, which he described as severe, and from slight hemorrhage. Only two bad results from the Mauser wounds were seen. One a comminuted fracture of the left radius, with subsequent necrosis of the bone, exfoliation of a sequestrum and union of the tendons of the radial side of the wrist. The other was one of our soldiers who was shot by a comrade who didn't know the gun was loaded. The bullet pierced the right chest and the man died from hemorrhage and shock in thirty minutes. Most of the other wounds seen weie in the extremities and the resultant scars were small round dots scarcely noticeable, the ball piercing the bone as neatly as a drill. These wounds indicated that the fighting had been done at over 400 yards.

Their weapons were of all manners of shapes and kinds and I have here a typical machete, presented by a Cuban officer, and it is of the usual model. It is a very useful weapon in a country where the undergrowth is so dense and so fine, as a road can be cleared in any place quickly with a weapon of this kind. As a weapon of warfare it was rarely used, except by the Cubans on their fellow countrymen whom they had captured fighting under the Spanish flag. This was done by slashing the victim across the abdomen and leaving him disemboweled to die. They never lived more than two days as the buzzards would complete what the Cubans had begun.

The poisonous insects were the tarantula and the scorpion. No cases of tarantula bites were seen and the Cubans did not know personally of any fatal case. The tarantula wound is slow to heal, painful and frequently followed by a neuritis along the nerve areas affected by the poison. Many of these creatures were captured and some cf them when stretched out would cover a circle whose diameter was seven inches. Scorpions were plentiful and the men were frequently stung, as every cactus hedge and stone pile is alive with them. Their bite is much like the common hornet sting; very painful at first, but giving slight inflammatory reaction and yielding rapidly to a carbolic dressing. Flies and mosquitoes are like the song birds, scarce.

Except in Havana, yellow fever is rarely seen during the winter months, unless a large body of troops with a natural susceptibility to it is encamped in a malarious region. This is rather interesting when we consider that each epidemic of recent years has been preceded by an outbreak of malarial fever, and yellow fever, so similar, clinically, to bilious remittent or pernicious malarial fever has followed these outbreaks. The Spaniards lost 7,000 men from yellow fever during the year 1897 and had established large fever hospitals at different places. On the edge of the harbor at Mariel was one of these hospitals with accommodations for 2,000 men, a sterilising plant, but no system of drainage or sewerage other than the slope to the harbor edge. All around the kitchens were piles of dried refuse, showing how careless they were in such matters.

On the 9th of December, 1898, the 202d regiment steamed into the harbor of Havana and unloading at the wharf took trains to the cities of Pinar Del Rio and Guanajay. During the first three weeks many of the men suffered from attacks of peri- and follicular tonsilitis as the result of exposure to the peculiarly damp, irritating night air. The dews were so heavy that by order the sentries on guard after dark wore their rubber ponchos to keep from being soaked, for otherwise their clothes would be as wet in the morning as if they had been standing in a heavy rain fall. The first cases of malaria occurred two days after a sudden rainfall, which left things an hour later steaming in the midday sun. These numbered about forty and each succeeding day saw new faces at sick call and new cases sent to the regimental hospital. Most of the cases during the month of January were tertian intermittent in type and were controlled by liberal doses of quinine. Recurrent attacks were more intractable and quinine had but slight effect. Warburg's tincture was tried faithfully, but the results were very unsatisfactory. Sodium salicylate, however, proved of immense value in cases associated with high temperatures and lengthened paroxysms. A commission sent through Cuba for the purpose of selecting camp-sites foi the regiments located us on a rolling country, beside a beautiful magnesia spring, but surrounded on two sides by marshy ground. While it was fully recognised that a large number of our malaria cases were dependent on the locality, yet we were not allowed to move to a better site until the quartermaster's department had concluded arrangements for the rental of other grounds, while in the meantime each day added to our sick list. In February the more dangerous forms of malaria were seen in the remittent types. In this form was seen the various complications not only troublesome and difficult to treat but dangerous to the life of the patient. About sixty men suffering from this form were transferred to the hospital ship Missouri, and from there to Savannah, where all but two recovered.

The complications most commonly seen were gastric disturbances, in which the patient would retch and vomit almost unceasingly for hours during the paroxysm; irritation of the intestinal tract with profuse diarrheas, resembling typhoid; cerebral irritation, in which the patient would be comatose or wildly delirious; involvement of the kidneys with acute congestion, suppression of urine and acute diffuse nephritis; bilious remittent, in which the patient became rapidly jaundiced and invariably called by the native doctors yellow fever. My tent mate, Dr. Wood, lost his life during his service in Cuba, having been taken suddenly one afternoon with shooting pains in the thighs, followed by a hard chill for two hours and a rise in temperature to 105° F. The next morning his temperature dropped to 101° F., with the appearance of a purpuric petechiæ, muttering delirium, small, rapid, irregular pulse, contracted pupils and occasional intervals of consciousness. He remained in this condition with the disappearance of the purpuric condition and an evening raise of one to two degrees for three weeks until a sudden paroxysm produced a serious apoplexy and death. Another fatal case was due to an acute diffuse nephritis, following the initial paroxysm and death following about five weeks later from uremia.

The most interesting case of all was one on which the experts disagreed and caused the report that the regiment was infected with yellow fever that subjected all to an unnecessary amount of worry which, had the men been less philosophic, would have been a bad thing for them, as they had little else to do than worry. A detachment of thirteen men and two officers was sent to the town of Mariel to guard government property there. Among these men was Private Clooney, Co. L. While off duty numerous excursions were made to the old fort and the fever hospital by the men and officers and after two weeks they were relieved by another detachment. Fifteen days after their return Private Clooney was seized with a severe chill, followed by a rise in temperature to 105° F., twenty-four hours later his temperature went up to 106°F. and he became jaundiced, had a slight albuminuria, diabetic breath, was in a state of profound coma, pulse rapid and small. On the third day experts from Havana diagnosticated the case as one of yellow fever from the presence of albumin in the urine, tenderness on pressure in the hypogastric region and the jaundice. This diagnosis was differed from by Dr. Howland of this city, then serving as regimental adjutant to the 202d, and by Dr. Lawrenson, yellow fever expert from New Orleans. The patient's temperature remained above 102½ until the eighth day, when he died. A post mortem revealed on opening the abdomen an enormously swollen spleen, liver swollen, dark slate colored, and smooth gall-bladder distended, but no evidences of gastro-intestinal involvement. The opinion of Drs. Howland and Lawrenson was confirmed in the official report, which states that while the first diagnosis was one of yellow fever the post mortem shows the case to have been one of pernicious malaria of the bilious remittent type. In speaking of the large number of malarial cases seen there it may be of interest to know that the mosquito played but small part in the propagation of this disease, as they were rarely seen and never troublesome.

During the month of December, 220 cases applied for treatment at sick call; in January, 5c5; in February, 509; in March, 343, and for the fifteen days in April, 37.

Furunculosis of the legs bothered the men in a good many instances. This was caused by the men scratching their legs with dirty nails for flea bites. A few of the cases were rather severe, but healed rapidly under appropriate treatment.

Streptococci and virulent infections were not seen, except in one case of tetanus in a two-year old child following a vaccination.

Following flea bites were cases of urticaria and three well-marked cases of dermatitis herpetiformis of the vesicobullous variety. Two recovered after five weeks rest in bed with tonics, and the like. The third case did not convalesce until he reached the cooler climate of Savannah, when his recovery became rapid.

The work laid out for our regiment was the establishment of order in the province, support to the native civil authorities, and lastly to try and teach them the value of municipal sanitation and to establish civil hospitals. At Guanajay, Mariel and surrounding towns the staff of doctors on duty vaccinated all the inhabitants, by house to house visits, who did not show a visible immunity to small-pox. Only one bad result was seen—that of the child mentioned a moment ago. On our arrival there were several cases of small-pox scattered around the towns and one morning a colored urchin, about twelve years of age, came calmly walking into the camp to watch the soldiers work, his face partially covered with the recent crusts of small-pox. The people do not regard isolation as necessary in these cases. The abandoned military hospital at Guanajay was thoroughly policed from top to bottom, rooms all washed, scraped and white-washed, grounds around the buildings cleaned and burned and then all the sick poor gathered in—about 120 in number. With the assistance of the mayor and chief of police all the prostitutes—thirty odd in number—were rounded up and examined. Eleven were placed in arrest in the civil hospital for treatment. Weekly examinations and keeping in camp the soldiers who were diseased kept the average of disability from venereal diseases lower during the stay in Cuba than it had been in this country. Usually from 2 to 4 per cent. were incapacitated from all duty from this cause, but owing to strict discipline and watchfulness, the percentage dropped to less than 1 per cent.

All latrines and privies in public and private dwellings were ordered cleansed and it was forbidden to throw any refuse into the street.

A street department was organised and paid for by the United States Government and all streets were cleansed and kept clean. At daybreak wagons called for the refuse and carted it three miles from the towns. All householders were urged to whitewash their houses outside and in and most of them did so. Stores particularly filthy and offensive were ordered cleansed and if this was not complied with they were shut up until they obeyed the order. One building was found full of old bedding, cots and dirty furniture. Inquiry developed the fact that it had been purchased a short time before from the yellow fever hospital and that it had never been disinfected. For this reason it was offered for sale at a very cheap figure. That building was closed up until the troops had left. It took the natives some time to understand that an order when issued must be obeyed as they had more faith in a five dollar gold piece than in any department order and they didn't get over this feeling until they saw that no one had a "pull" with the authorities.

The greatest danger to troops serving in that country by far is from malaria, as there is no immunity and as our soldiers are particularly susceptible to it and one attack predisposes to another, one can easily see what continued service for a regiment would do. Certain general principles, as carried out by the British government in the Island of Jamaica, would render life tolerable there, but if the troops forming the army of occupation are given only the scanty shelter they have had so far, we may look for a large death-rate among the men for the next year. After nine and a half months' service the 202d comes home with the following record: The camp was always clean; cleaner than the camps of the other volunteer regiments that we were thrown in contact with, as the records of the field officers of the day for the 2d Army Corps will show. Special attention was always paid to the company kitchens and the cooking utensils, sinks and the company streets; and with a clean camp from the time we started in until the regiment was mustered out we have the following mortality record: Deaths—from suicide, 1; from railroad accident, 1; from gunshot injuries, 2; from malaria and its effects, four; and from typhoid fever, 8.

90 North Pearl Street.

